# Fostering Inclusive Excellence: Reimagining Oral Science Research and Admissions to Advance Dentistry During Perilous Times

**DOI:** 10.1002/jdd.70024

**Published:** 2025-09-19

**Authors:** RK, JB, YK, DG

**Affiliations:** ^1^ Higher Education Institutions Across the United States

**Keywords:** Dental school admissions, dental, diversity, education, equity, health equity, inclusion, systemic racism

## Abstract

**Purpose**: Recent shifts in public policy and legislation have aimed to dismantle progress toward racial equity in the United States, especially within the realm of education. Dental education institutions are responsible for cultivating the oral healthcare workforce of the future, but their ability to meet the growing dental burden is hampered by the intentional dismantling of equitable, race‐conscious policies in education. This manuscript makes the case for dental school administrators and faculty to ethically promote racial equity and inclusion in their institutions and proposes strategies to accomplish this goal.

**Methods**: The authors synthesized existing evidence to support the promotion of anti‐racist initiatives in oral health education and dental curricula. They proposed a three‐pronged call to action detailing strategies that dental school administrators could rapidly implement to drive equity and higher‐quality dental education and oral healthcare more broadly.

**Results**: The authors strongly recommend that dental school administrators: 1) Intentionally frame a message of urgency regarding the promotion of racial equity in dental education, 2) Elevate student leaders to be the leading advocates for inclusivity in dental schools, and 3) Evaluate student applicants holistically using professional competencies, so the future dental workforce will feature professionals who uphold all the standards of the profession, not just academic standards.

**Conclusions**: The reversal of racially inclusive educational policies threatens to diminish the quality of dental education. With these proposed strategies and others, educators can improve dental schools nationally, and the future oral healthcare workforce can better serve all people.

## 1 Introduction

Racism is an organized social system of advantage based on race and is used to disempower and differentially allocate valued resources and opportunities to groups deemed inferior [1, 2, 3]. Race itself is a socially constructed concept, shaped by racialization—the processes through which social meaning is assigned to groups based on physical or social characteristics like nationality, culture, and phenotype; these are falsely assumed to reflect inherent biological differences [4, 5]. This process is inextricably linked to the ideology of racial hierarchy, wherein racial groups are ranked as superior or inferior [6, 7].

Racial beliefs and hierarchies are transmitted through socialization, which begins in childhood and is reinforced by institutions and culture [8]. While racism is often associated with overt acts of prejudice and bias, it operates most pervasively at the structural level through racialized laws, policies, and practices that uphold white supremacy and perpetuate the oppression of racial groups deemed inferior [2, 4]. In the United States (US), structural racism was foundational to the nation's colonial–settler development and was codified through laws and policies across multiple sectors of society, giving rise to legal and tacit systems and structures of racial oppression (e.g., Jim Crow laws) [9, 10]. Though civil rights legislation sought to end de jure discrimination [4], structural racism persists today through both explicit and race‐neutral policies that continue to reinforce a racially hierarchical social structure, maintaining and perpetuating the relational subordination of people along ethnoracial lines [11].

The consequences of structural racism extend beyond economic and social inequities, profoundly shaping health outcomes—including oral health. Despite major improvements in oral health over the past 50 years, stark disparities persist globally and in the U.S [12, 13]. The historical consequences of structural racism, such as residential segregation, have led to the maldistribution of dental providers, limited access to affordable dental services, and differential exposure to environmental risk factors, all which contribute to poorer oral health outcomes for ethnoracial minoritized populations [14]. While some systemic conditions are rooted in intentional policy decisions, others continue due to inaction, requiring intentional systemic reevaluation and transformative action. Importantly, inequalities in access to dental care have been implicated as a key driver of oral health disparities, rather than individual behaviors such as hygiene or diet, and these broad systemic drivers are still shaping oral health outcomes longitudinally [12, 15, 16].

Despite a growing collective understanding surrounding the impact of structural racism on oral health, the extent to which historically racist systems and structures continue to shape dentistry and oral science research remains underexplored. In addition to existing disparities in access to care, other factors—such as medical mistrust, historical discrimination, and insufficient recruitment of underrepresented ethnoracial minoritized groups into the dental and oral science workforce—exacerbate the consequences of structural racism in dentistry, ultimately leading to poorer health outcomes for minoritized populations [17, 18].

### 1.1 Promoting Anti‐Racism in Oral Health Education and Admissions

As the US experiences a period of retrenchment—marked by resistance to racial progress and the rollback of equity initiatives—it is more critical than ever for academic institutions to explicitly embed anti‐racism in their mission statements. The 2023 reversal of Affirmative Action has exacerbated challenges in fostering diverse educational environments, despite extensive research demonstrating that interacting with individuals from different racial or ethnic backgrounds enhances leadership skills, critical thinking, and civic engagement. Notably, a diverse student body has been shown to have a greater impact on these skills than a diversity‐focused curriculum alone [19]. Given that the erosion of race‐conscious policies threatens the quality of predoctoral dental education, institutions must reaffirm their commitment to diversity and inclusion as foundational values. One way to advance this commitment is through the intentional use of anti‐racism pedagogy, a framework grounded in Critical Theory that seeks to explain and counteract the persistence and impact of racism [20]. Its core elements include: consciousness (awareness and understanding of how society operates with regard to race), praxis (reflection and action that lead to antiracist behaviors), and a critical perspective of a racist society (interrogating how racial formations shape institutions and experiences). Some research‐intensive dental schools have taken proactive steps by maintaining diversity as a central pillar of their recruitment strategies, demonstrating that institutional commitment can sustain progress even in the face of restrictive policies.

Beyond fostering a diverse environment, predoctoral dental education institutions must confront the historical and systemic drivers of oral health inequities. An anti‐racist curriculum should critically examine how structural racism intersects with other systems of oppression—such as sexism and classism—to shape health disparities, including in oral health [21]. The long history of scientific and medical exploitation in the US—from the unauthorized use of Henrietta Lacks’ cells to the Tuskegee syphilis study to the Stateville Penitentiary malaria experiments—has fueled deep‐rooted medical mistrust among intersectionally marginalized communities [22, 23, 24]. This mistrust continues to influence health‐seeking behaviors and treatment outcomes today [17, 25], necessitating open discussions about these histories as part of predoctoral dental education. In a climate where institutions may face pressure to dilute or eliminate race‐conscious discussions, dental schools have an ethical responsibility to resist this regression. Because many academic institutions historically played a direct role in medical exploitation and exclusionary practices, they have a moral obligation to confront these legacies and actively work towards dismantling their harmful consequences.

### 1.2 Call to Action

### 1.3 Values Don't Change, Framing Does

In times of uncertainty and retrenchment, oral science research and academic dentistry cannot afford complacency. Meaningful progress requires bold, courageous leadership—leaders who are unwavering in their commitment to equity, innovation, and the public good. Leadership rooted in a strong moral compass is essential to navigating these challenges, ensuring that the core values of academic dentistry remain steadfast despite external pressures. The pursuit of justice, inclusion, and excellence in predoctoral dental education and healthcare must not waver in the face of financial constraints or institutional setbacks. Now, more than ever, leaders must embrace transformative action and advocate for policies and practices that advance equity and access to dental care.

However, leadership alone is not enough—real change is driven by the collective power of an engaged academic and research community. One of the greatest challenges is crafting a compelling narrative that conveys the urgency of our mission. A systems view of oral health, rooted in community and shared responsibility, often runs counter to the dominant cultural narrative of rugged individualism. Yet, the language of community—which emphasizes interconnectedness, reciprocity, and collective responsibility—must drive our movement forward. To achieve this, we must reframe our messaging, making explicit the moral urgency of oral health equity and ensuring that those most affected are at the forefront of shaping these efforts. The power of framing cannot be underestimated—how we communicate our mission determines whether it resonates, mobilizes, and leads to real systemic change. Retrenchment must not mean regression; rather, it is an opportunity to reassert our values, strengthen our collective resolve, and co‐create a future where academic dentistry and oral science research remain a force for justice, health, and social progress.

### 1.4 Foster Student Leadership, Empower Student Voices

As academic dentistry navigates an era of retrenchment, cultivating meaningful student leadership is more critical than ever. Ubiquitously, dental schools prioritize students in their mission and vision statements, often describing students as the next generation of leaders in the field and the future of our dental workforce. Consequently, elevating student voices may seem like a natural and widely practiced concept. However, without intentional efforts to actively seek, validate, and respond to student input through systemic, administrative action, the full benefits of student leadership and diverse perspectives cannot fully be realized. There are various players in administrative roles, including Deans, Associate/Assistant Deans, Department Chairs, and members of curriculum and admission committees. With the layered hierarchies in dental schools, it is essential that all administrators are committed to elevating student voices and providing platforms and resources for students to easily navigate such expression. Intentional structures, such as compensated student advisory boards, dedicated mentorship pathways, and voting roles on curriculum committees, are necessary to move beyond performative engagement and into shared power.

Overcoming systemic inequities in oral healthcare and dental admissions and preparing a workforce capable of addressing the nation's oral healthcare needs requires a diverse student body. However, recent policy shifts have made it increasingly difficult for administrators to openly champion diversity, equity, and inclusion within their institutions and admissions practices. In this landscape, students can play a pivotal role in advocating for these values, often with a level of flexibility and grassroots influence that institutional leaders may lack. By fostering mentorship structures, supporting student‐led initiatives, and equipping student leaders with tools to engage in policy discussions, dental schools can help amplify their voices at both institutional and national levels. Virtual meetings can enable students nationwide to collaborate with administrative support and engage with pre‐dental students, expanding the movement for inclusivity.

A prime example of student‐driven impact can be seen at a research‐intensive institution in California, where student advocacy led to sustainable clinical practices, reducing waste and saving thousands of dollars in disposal costs [26]. This illustrates how student leadership, when supported by an institutional infrastructure, can drive meaningful and lasting change. Without diversifying and training faculty, structural racism will be reproduced across generations of students. Thus, it is essential that students trained in anti‐racist practices continue to be supported throughout their careers, including the implementation of faculty development programs grounded in anti‐racism pedagogy and equity‐centered education and fellowships to support historically excluded dental students in pursuing academic careers. In an era where academic dentistry must defend and redefine its values, empowering students is not just an investment in the future of the profession; it is a strategic necessity for advancing equity, innovation, and systemic progress.

### 1.5 Emphasize Holistic Admissions With Professional Competencies

In pursuing a more equitable and socially responsible dental profession, admission practices must reflect the values of diversity, inclusion, and fairness. Traditional admissions criteria—such as grade point average and standardized test scores—often serve as proxies for privilege, reflecting access to resources (e.g., costly test preparation, stable living conditions, and prior educational experiences) rather than true potential or ability [27]. Over‐reliance on numerical metrics can inadvertently favor more privileged applicants while overlooking candidates who have demonstrated adaptability, perseverance, and leadership in adversity. To cultivate compassionate dentists and leaders, we encourage admissions committees to utilize a holistic review that assesses qualities such as resilience, dedication, and community engagement—traits crucial for future dental professionals.

Despite increasing recognition of holistic admissions in medicine, dentistry lags behind in establishing standardized competency‐based criteria for evaluating applicants. The Association of American Medical Colleges (AAMC) aptly provides a comprehensive framework that assesses applicants across professional, science, and thinking and reasoning competencies, last updated in 2023 [28]. Surprisingly, no equivalent system exists for dental school admissions. Historically, the ADEA has explored holistic admissions approaches, factoring in non‐academic qualifications like extracurricular activities, volunteer work, research experience, and first‐generation educational status [29]. In addition, the ADEA Admissions Committee Workshop, spanning from 2004 to 2009, provided predoctoral dental schools with guidance surrounding recruitment and admissions practices, emphasizing holistic admissions and interprofessional collaboration [30, 31]. These strategies, along with the AAMC's current competencies and core framework, encourage a holistic approach to admissions and student evaluation, recognizing that a numbers‐driven admissions process fails to capture essential qualities that yield successful dentists. A competencies‐based approach ensures that admissions committees evaluate the full spectrum of an applicant's potential, including experiences in overcoming personal adversity, participation in research, leadership in community‐based initiatives, and commitment to service. These experiences highlight the candidate's capacity to become a well‐rounded, compassionate, and culturally competent and humble provider who can connect with and treat a diverse patient population, an ability that is prioritized in existing Commission on Dental Accreditation (CODA) standards for predoctoral dental education [32, 33]. In addition to considering students’ competencies, admissions executives ought to consider their admitted class as a whole, rather than a collection of individual applicants, so students with different strengths and experiences can come together for a more impactful educational experience and learning environment.

Dentistry is not only a field based on technique—it is a profession that requires strong interpersonal skills, ethical decision‐making, and a deep understanding of human experiences. By embracing holistic admissions, dental schools can cultivate a generation of dentists who are competent clinicians and compassionate leaders. We encourage the American Dental Education Association (ADEA) to prepare a professional competencies list similar to AAMC's for dental schools. In the short term, dental admission committees should consider adopting AAMC's competencies and embracing CODA accreditation standards as a guide to reshape their admissions strategies.

## 2 Conclusion

Oral health equity is a fundamental commitment to justice and well‐being. Retrenchment must not mean regression—rather, it must ignite a renewed commitment to our values, a collective resolve to shape a future where oral science research and academic dentistry stand as beacons of justice, health, and social progress. Student leadership is the driving force behind transformative change, and their voices must be empowered, not silenced. Holistic admissions is not just an alternative approach; it is a necessary evolution that recognizes the full spectrum of human potential. And how we frame our message—how we communicate the urgency of this moment—will determine whether our movement flourishes or falters. Now is the time to act with courage, to reimagine a more just and inclusive oral science research landscape and dental profession, and to refuse to let structural barriers dictate the future of dental care. The responsibility is ours—what role will you play?

## Conflicts of Interest

The authors declare no conflicts of interest.
